# A potential surrogate for poliovirus in testing the efficacy of hand antiseptics according to the Global Poliovirus Containment Action Plan 

**DOI:** 10.3205/dgkh000568

**Published:** 2025-07-09

**Authors:** Maren Eggers, Katharina Konrat, Nils Hübner, Ingeborg Schwebke

**Affiliations:** 1 Labor Prof. Gisela Enders MVZ GbR, Stuttgart, Germany; 2 Robert Koch-Institute, Berlin, Germany; 3 University Medicine Greifswald, Institute for Hygiene and Environmental Medicine, Greifswald, Germany

**Keywords:** poliovirus containment, picornaviridae, encephalomyocarditis virus, virucidal efficacy, disinfectant testing, surrogate virus, quantitative suspension test

## Abstract

**Introduction:**

As part of the global poliovirus containment program (Global Polio Eradication Initiative, GPEI), European laboratories are increasingly restricted from using the long-established poliovirus type 1 strain LSc-2ab as the reference virus in virucidal efficacy testing of disinfectants.

This necessitates the identification of an alternative test virus that closely resembles poliovirus in its chemical resistance, belongs to the picornaviridae family, and is suitable for routine laboratory handling.

**Materials and methods:**

In this study, two strains of encephalomyocarditis virus (EMCV) were evaluated as potential substitutes in quantitative suspension tests (according to EN 14476) using five biocidal active substances: ethanol, propan-1-ol, propan-2-ol, glutaral (glutaraldehyde) and peracetic acid.

**Results:**

The results demonstrated a strong correlation between the inactivation profiles of EMCV and Sabin poliovirus type 1 vaccine strain LSc-2ab. Due to this close correlation and EMCV’s practical advantages in laboratory settings such as availability in a European virus bank, growth in a high titer, it is proposed as a suitable substitution candidate for future virucidal efficacy testing.

## Zusammenfassung

**Einleitung:**

Im Rahmen der globalen Polioeradikationsinitiative (Global Polio Eradication Initiative, GPEI) werden die europäischen Laboratorien hinsichtlich der Verwendung des seit Jahrzehnten etablierte Poliovirus Typ 1, Stamm LSc-2ab als Referenzvirus für die Prüfung der viruziden Wirksamkeit von Desinfektionsmitteln zunehmend eingeschränkt. Daraus ergibt sich die Notwendigkeit, ein alternatives Testvirus zu identifizieren, das dem Poliovirus in seiner chemischen Resistenz sehr ähnlich ist, zur Familie der Picornaviridae gehört und für die routinemäßige Handhabung im Labor geeignet ist.

**Material und Methode:**

: In dieser Studie wurden zwei Stämme des Enzephalomyokarditis-Virus (EMCV) als potenzielle Ersatzkandidaten in quantitativen Suspensionstests (gemäß EN 14476) unter Verwendung von fünf bioziden Wirkstoffen bewertet: Ethanol, Propan-1-ol, Propan-2-ol, Glutaral (Glutaraldehyd) und Peressigsäure.

**Ergebnisse:**

Die Ergebnisse zeigten eine sehr gute Korrelation zwischen den Inaktivierungsprofilen von EMCV und Sabin-Poliovirus Typ 1-Impfstoffstamm LSc-2ab. Aufgrund dieser Übereinstimmungen sowie der unkomplizierten Laborhandhabung von EMCV, wie z. B. der Verfügbarkeit in einer europäischen Virusbank und dem Wachstum in hohen Titern, stellt EMCV einen geeigneten Ersatzkandidat für zukünftige viruzide Wirksamkeitstests dar.

## Introduction

As a result of the Global Polio Eradication Initiative, poliovirus cases have declined by more than 99% since 1988. The global transmission of both wild poliovirus type 2 (WPV2) and wild poliovirus type 3 (WPV3) has been successfully interrupted. The last reported cases of these viruses occurred in 1999 and 2012, respectively [1]. In addition, Poliovirus type 1 continues to be only sporadically transmitted in endemic areas and therefore further laboratory use of poliovirus type 1 will be restricted according to the World Health Organization (WHO) Polio Eradication Initiative and containment measures, especially as humans are the only known reservoir for poliovirus [2]. However, Sabin poliovirus type 1 vaccine strain LSc-2ab (PV-1) has been most widely used for standardized virucidal testing for decades. As PV-1 is a non-enveloped virus [3], it is very resistant to biocides [4], [5]. In addition, a virucidal activity against PV-1 also includes efficacy against the most clinically relevant picornaviruses [6], such as the causative agent of hand-foot-and-mouth disease, enterovirus Human enterovirus 71 (EV-71), and Coxsackie A viruses (A2 - A8, A10, A12, A14, A16) [7]; Enterovirus D68, a pathogen that can induce acute flaccid paralysis similar to that caused by poliovirus, is also included in this virucidal claim [8]. Since 2024, the presence of circulating vaccine-derived poliovirus type 2 (cVDPV2) was detected in wastewater samples collected from Finland, Germany, Poland, Spain and the United Kingdom (UK) [9]. This finding indicates the potential for risk of infection to vulnerable persons in Europe and emphasizes the need for ongoing monitoring and preventive measures such as disinfection. Consequently, disinfectants and antiseptics must be able to prevent the transmission of Vaccine derived poliovirus type 2. 

However, the goal of the Global Poliovirus Containment Action Plan (GPCAP) 2022–2024 [10] is to minimize the number of facilities holding poliovirus material, and many European countries have implemented this by no longer allowing work with poliovirus outside of a designated poliovirus essential facility (PEF). Thus, a standardized surrogate virus that is equally resistant as the poliovirus is needed for European efficacy testing. The surrogate virus must as easy be propagated in high titers as the PV-1, must be available in virus collections, and must have a low biosafety level [6]. Preferably, it should also belong to the picornavirus family and be available in a European virus bank. In this study, a comparison was made between two encephalomyocarditis viruses (EMCV), which belongs to the genus cardiovirus in the picornaviridae family, and the PV-1. EMCV is a small non-enveloped single-strand RNA virus and the causative agent of not only myocarditis and encephalitis, but also neurological diseases, reproductive disorders and diabetes in some mammalian species [11]. EMCV has already been used as a surrogate virus for various non-enveloped viruses in several studies [12], [13], [14], [15]. Supplementary Table S1 in Attachment 1 presents details that facilitate a more thorough examination of these data.

In the present study, the virucidal efficacy of ethanol, propan-1-ol, propan-2-ol, glutaral (1,5-Pentandial, glutaraldehyde, GDA) and peracetic acid (PAA) was evaluated against two representatives of these cardioviruses – EMCV strain Cuba (EMCV-C) and EMCV strain Ungarn (EMCV-U), and the results were compared to the virus reduction obtained in parallel with the current test virus, PV-1. 

## Material and methods

### Test viruses and virus propagation 

EMCV (encephalomyocarditis virus, kindly provided by Friedrich Loeffler Institute (FLI), Insel Riems, Germany) strain Cuba (EMCV-C) and strain Ungarn (EMCV-U) were propagated in BHK-21 cells (DSMZ) under use of Dulbecco’s modified Eagle’s medium (DMEM, 4.5 g/L glucose, Gibco). The infected cell culture flask was incubated at 37°C and 5% CO_2_ until 80–90% of the cells showed a cytopathic effect (after 1–2 days). The cells were frozen and thawed once, followed by centrifugation at 5,000 rpm for 15 minutes. The supernatant was aliquoted as test virus suspension and stored at –80°C. The virus titers were in the range of 10^9^ tissue culture infectious dose 50% (TCID_50_)/mL. 

### Propagation of Sabin poliovirus type 1 vaccine strain LSc-2ab (PV-1) 

In laboratory 1 poliovirus type 1 (Sabin original [LSc-2ab]) from the WHO (National Institute for Biological Standards and Control (NIBSC) code: 16/196) was grown in BGM cells (FLI) using Dulbecco’s modified Eagle’s medium (DMEM, 4.5 g/L glucose, Gibco). This strain was provided by the National Reference Center and the Regional WHO/EURO Reference Center for Poliomyelitis and Enteroviruses at the Robert Koch Institute (RKI). The infected cell culture flask was incubated at 37°C and 5% CO_2_ until 80–90% of the cells showed a cytopathic effect (after 3–4 days). The cells with supernatant were centrifuged at 6,000 rpm for 10 minutes by 4°C. After a threefold freeze/thaw procedure, the supernatant was ultra-centrifuged at 19,000 rpm for 4 hours by 4°C. The sediment contains the virus and was dissolved like aliquoted in serum free medium (stored at –80°C). The virus titer was in the range of 10^10^ TCID_50_/mL 

In laboratory 2 poliovirus type 1 strain LSc-2ab (Sabin original [LSc-2ab]) manufactured by Chiron Behring, Marburg (Supply source Eurovir, Luckenwalde, Germany) was propagated in BGM cells (FLI) under use of Eagle’s medium (EMEM, 4.5 g/L glucose, BioSell). The infected cell culture flask was incubated at 37°C and 5% CO_2_ until 80–90% of the cells showed a cytopathic effect (after 3–4 days followed by a threefold freeze/thaw procedure). The supernatant was removed and aliquots of the test virus suspension were stored at –80°C.

### Concentration and contact times in biocide testing 

The concentration of a product test solution was 1.25 times the desired test concentration, as the test product is diluted to 80% in the quantitative suspension test except tests performed with propan-1-ol and propan-2-ol (see Table 1 (Tab. 1)). 

Ethanol (≥99.8% (GC), for molecular biology 99.8%) was obtained from Sigma/Aldrich (ref. 1.08543.0250). The following concentrations were tested with an exposure time of 1 minute: 50%, 60%, 70% and 80% (w/w). In addition, 70% (w/w) was tested with exposure times of 5, 15 and 30 minutes. Propan-1-ol (≥99.5% for analysis EMSURE^®^ ACS, Reag. Ph Eur) and propan-2-ol (≥99.8% (GC), ACS reagent, reag. Ph. Eur., reag. ISO, EMSURE^®^) were obtained from Sigma/Aldrich (ref. 100997.1000 or 1.09634.1000). The following concentrations of both propanols were tested with an exposure time of 60 minutes: 70%, 80% and 90% (v/v).

Testing solutions of PAA and GDA to be tested for virus inactivation were prepared from commercially available products as previously published [16]. Lerasept special (5 g/100 g PAA) was obtained from Stockmeier Chemie GmbH & Co. KG (D-33609 Bielefeld). A 1.25% stock solution was prepared immediately prior to testing in all participating laboratories by adding 11.16 mL Lerasept spezial to 50 mL water of standardized hardness (WSH). Then, the solution was diluted to 0.005%, 0.01%, 0.02%, 0.05%, 0.075% and 0.080% (v/v) with WSH. The GDA-based antimicrobial product Protectol^®^ GA 50 was supplied by BASF, Ludwigshafen, Germany. Before each test, a 1.25% stock solution was prepared by adding 2.21 mL of the solution to 100 mL of WSH. This solution was then further diluted to 0.01%, 0.05%, 0.1%, and 0.25% (v/v). The contact time was 30 minutes for PAA and GDA, respectively (see Table 2 (Tab. 2)).

### Quantitive suspension test 

Both EMCV strains and PV-1 were tested with the 5 biocides in different approaches at 20°C according to the European Standard EN 14476 [17]. Briefly, one part by volume of the test virus suspension and one part by volume of interfering substance were mixed with eight parts by volume of the different biocide concentrations. Water was used as interfering substance and in the control test instead of the alcohols. The interfering substance for clean conditions used in the tests with PAA and GDA was 0.03% bovine serum albumin (BSA). The cytotoxicity was additionally determined. At the end of the chosen exposure time, the activity of the biocide was stopped by serial dilution with ice-cold medium.

The virus titers were determined using the Spearman and Kaerber method and expressed as lg TCID_50_/mL. The virucidal activity was determined by the difference of the logarithmic titer of the virus control minus the logarithmic titer of the test virus (lg TCID_50_/mL). According to DIN EN 14476 [17], a reduction in infectivity of 4 lg steps (99.99% inactivation) is considered sufficient to demonstrate virucidal activity. All tests were performed at least three times in two laboratories. 

Statistical analysis and data visualization were performed using GraphPad Prism 9 for Windows (GraphPad Software, San Diego, CA, USA). In order to analyze the correlation between reduction factors of PV-1, EMCV-U, and EMCV-C in two laboratories, one-way analysis of variance (ANOVA) was performed. One requirement for the application of ANOVA is the homogeneity of observed variances, which was tested using the Bartlett-test.

## Results

Two strains of EMCV (EMCV-U, EMCV-C) and the PV-1 were analyzed in suspension tests according to EN 14476. 

### Time dependence when exposed to ethanol 

**Figure 1:**
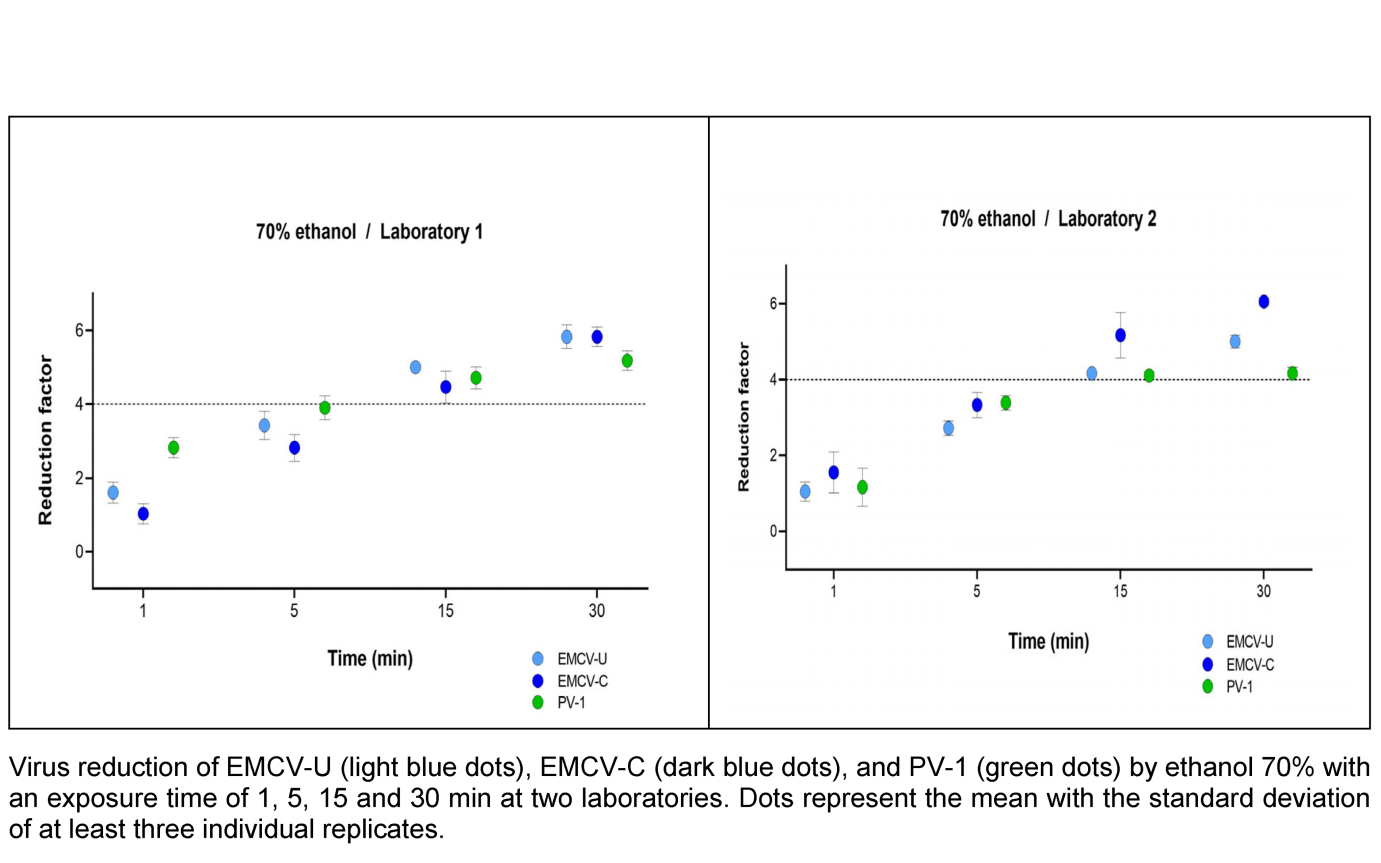
Contact time-dependent kinetic of ethanol 70% (w/w) against EMCV and PV-1

The time dependence of the reduction by ethanol exposure was investigated at a concentration of 70% (w/w). Exposure times of 1, 5, 15 and 30 min were tested. In both laboratories, efficacy was achieved within an exposure time of 15 min. At 5 min, the reduction was less than 4.0 lg for all viruses (Figure 1 (Fig. 1)). The differences between the two cardioviruses (EMCV-U, EMCV-C) and PV-1 are only slight and are not significant. 

### Concentration dependence of the reduction when exposed to ethanol 

**Figure 2:**
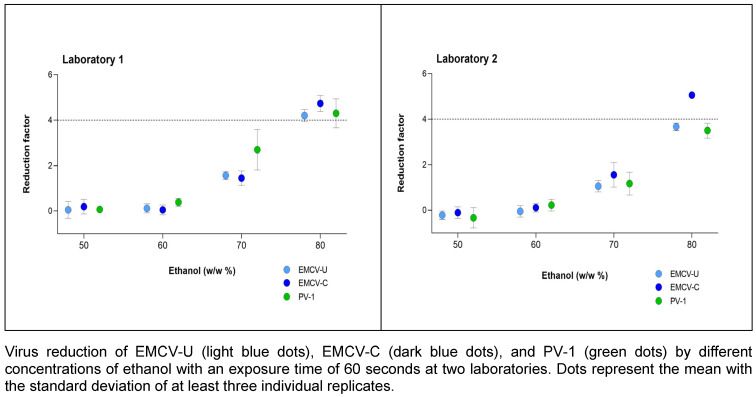
Concentration-dependent kinetic of ethanol against EMCV and PV-1

Ethanol was used as 50%, 60%, 70% and 80% (w/w) solution with an exposure time of 1 min. Both laboratories obtained good agreement. Figure 2 (Fig. 2) shows that laboratory 1 achieved a 4.0 lg reduction with 80% (w/w) ethanol for all three viruses. In laboratory 2, this was only achieved for EMCV-C. EMCV-C proved to be the most sensitive strain in both laboratories. Nevertheless, PV-1 and EMCV-U demonstrated nearly identical values for the majority of concentrations.

### Reduction due to exposure to propan-1-ol and propan-2-ol 

Both EMCV strains could not be inactivated by propan-1-ol and propan-2-ol (EMCV-U data of four replicates see Table 3 (Tab. 3) and Table 4 (Tab. 4), EMCV-C data not shown). 

### Reduction due to exposure to PAA 

**Figure 3:**
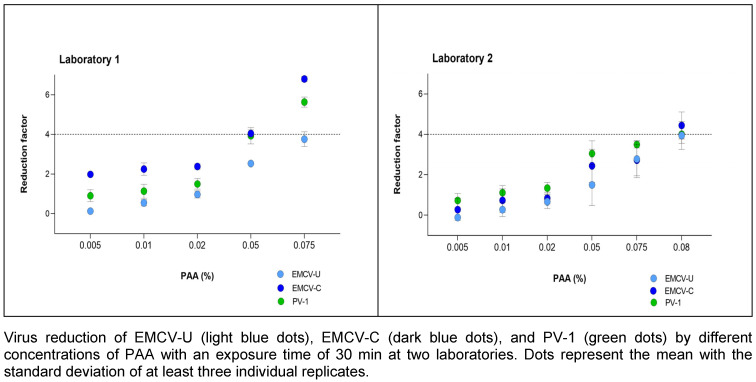
Concentration-dependent kinetic of PAA against EMCV and PV-1

PAA was tested at concentrations of 0.005, 0.01, 0.02, 0.05, 0.075 and 0.08% (v/v) with a fixed exposure time of 30 minutes. In laboratory 1, both EMCV strains are more sensitive at concentrations of 0.075% (v/v) PAA and the reduction is higher than in laboratory 2. The effective range (4.0 lg) was not reached until the concentration was increased to 0.08% (v/v) in laboratory 2 (Figure 3 (Fig. 3)).

### Reduction due to exposure to GDA 

**Figure 4:**
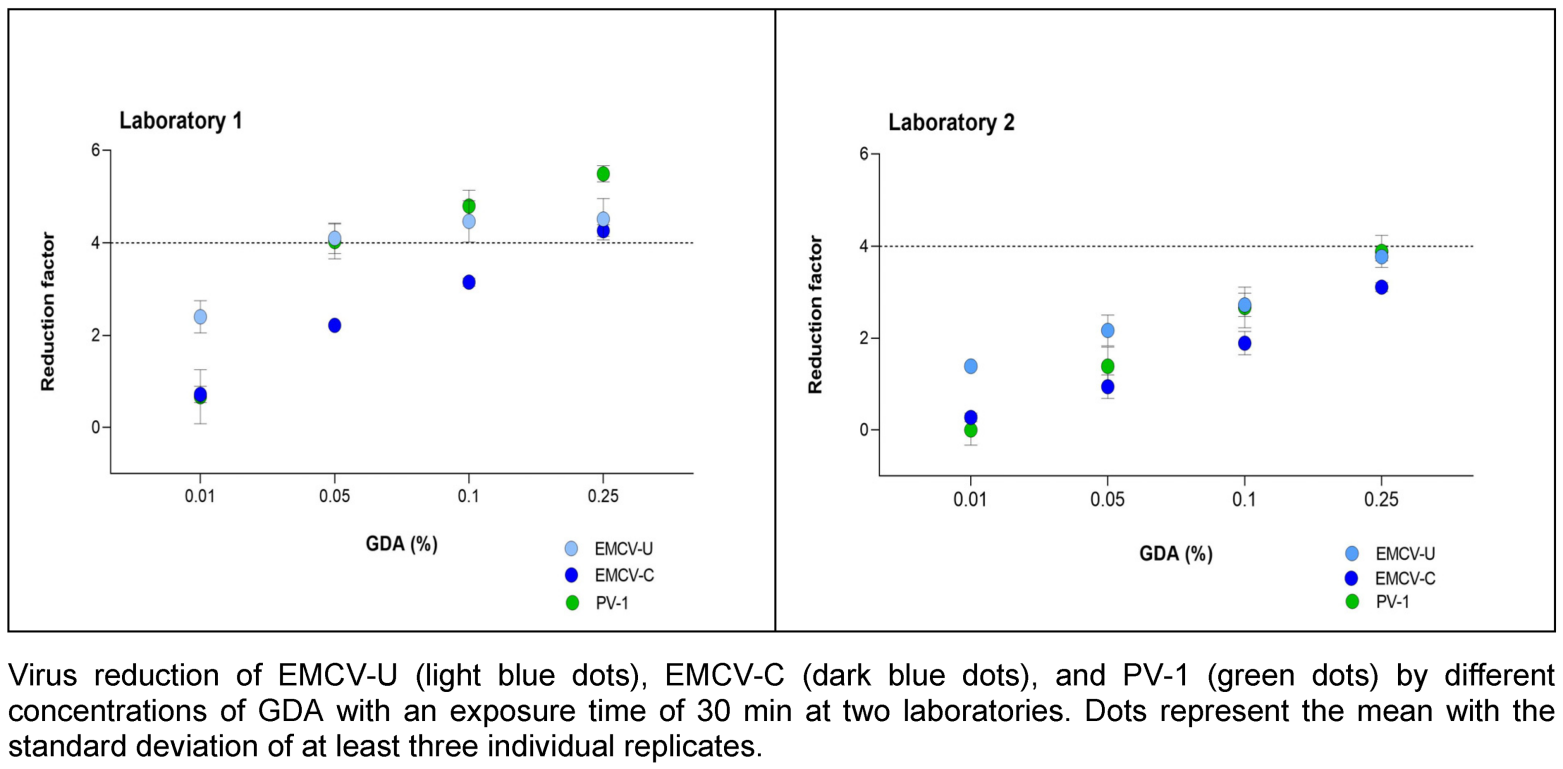
Concentration-dependent kinetic of GDA against EMCV and PV-1

GDA was tested at concentrations of 0.01, 0.05, 0.1 and 0.25% (v/v) with a fixed exposure time of 30 minutes. With GDA, EMCV-C proved to be the more resistant strain. In laboratory 1, a reduction of >4.0 lg was achieved with EMCV-U at a concentration of 0.05% (v/v), while a reduction of 4.0 lg was only achieved with EMCV-C at a concentration of 0.25% (v/v) (Figure 4 (Fig. 4)). In laboratory 1, the reduction of EMCV-U and PV-1 exhibited a high degree of similarity. In laboratory 2, the reductions are smaller, but the results of the three test viruses do not differ significantly.

### Correlation analysis 

**Figure 5:**
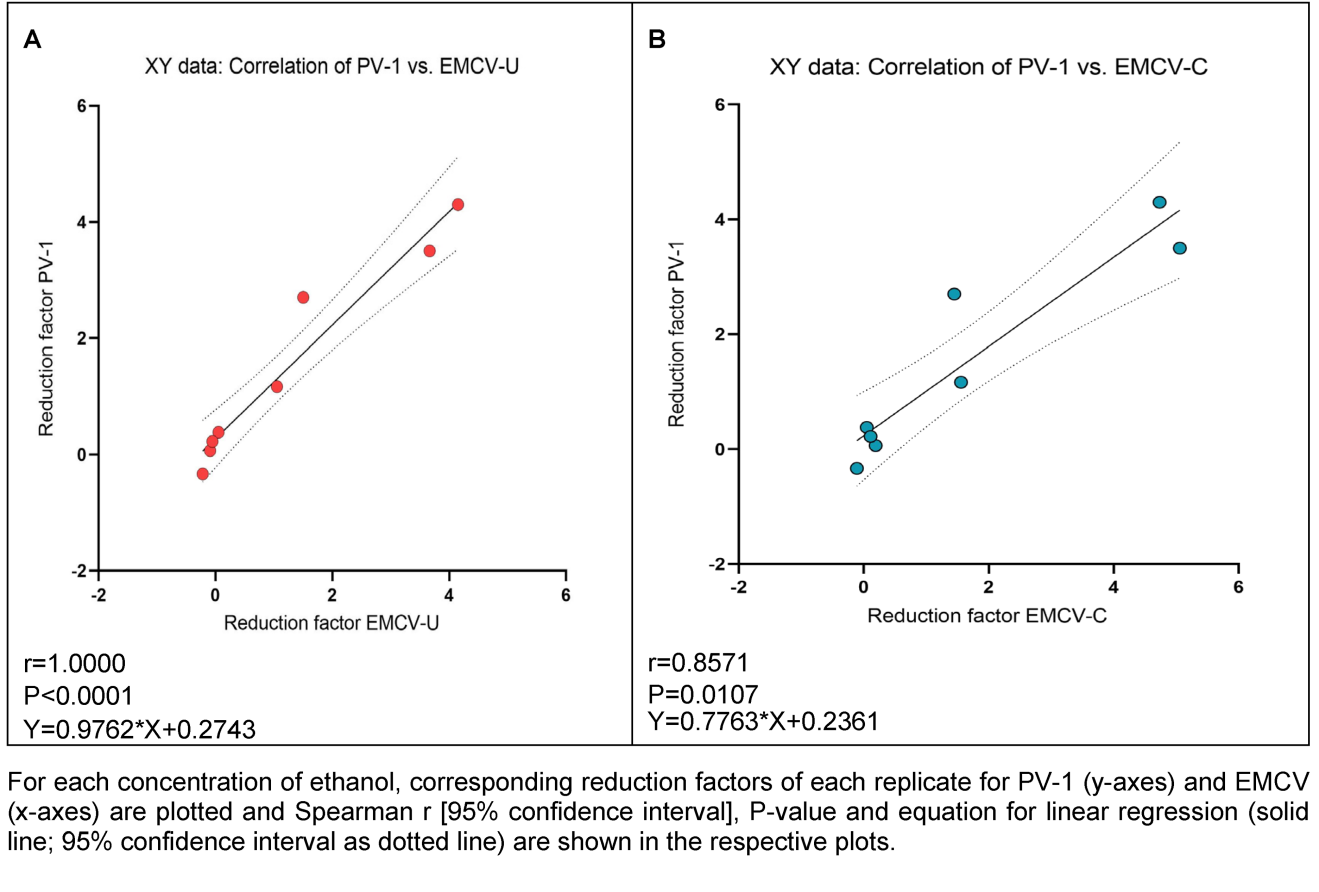
Correlation of reduction factors of PV-1 and EMCV-U (A), and PV-1 and EMCV-C (B) by ethanol

The maximum correlation was found between PV-1 and EMCV-U (Spearman correlation coefficient r=1.0000) (Figure 5A (Fig. 5)). However, a robust correlation has been observed between PV-1 and EMCV-C (r=0.8571) (Figure 5B (Fig. 5)).

**Figure 6:**
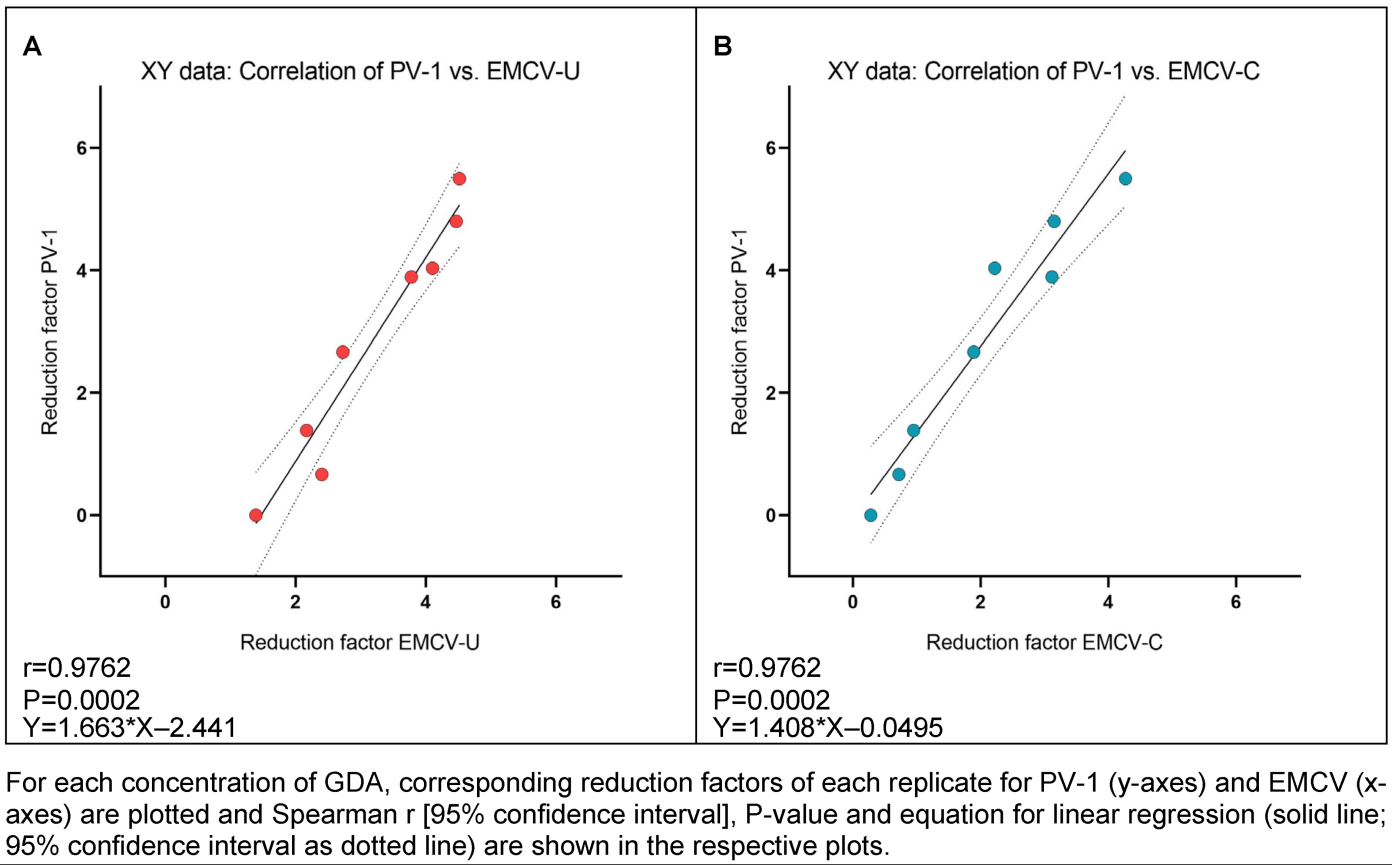
Correlation of reduction factors of PV-1 and EMCV-U (A), and PV-1 and EMCV-C (B) by GDA

**Figure 7:**
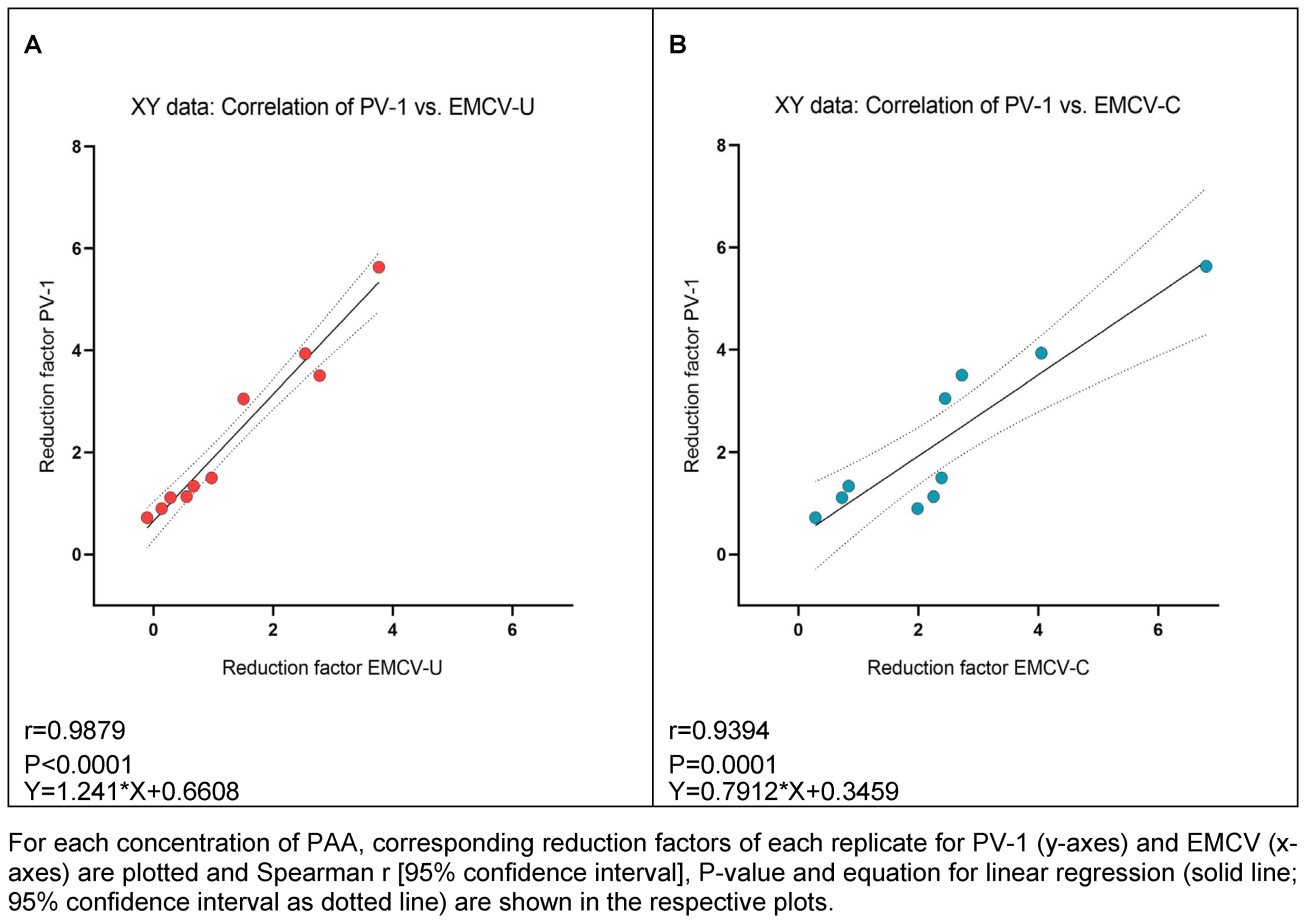
Correlation of reduction factors of PV-1 and EMCV-U (A), and PV-1 and EMCV-C (B) by PAA

As indicated by the high correlation values between PV-1 and both EMCV strains for GDA and PAA, there appears to be a strong correlation of PV-1 and EMCV-U reduction factors for GDA (r=0.9762; Figure 6A (Fig. 6)) and PAA (r=0.9879; Figure 7A), as well as between PV-1 and EMCV-C reduction factors for GDA (r=0.9762; Figure 6B (Fig. 6)) and PAA (r=0.9394; Figure 7B (Fig. 7)).

## Discussion

Polioviruses have long served as a benchmark in evaluating the efficacy of disinfectants and antiseptics due to their exceptional resistance to chemical inactivation. However, in light of the Global Polio Eradication Initiative and increasing biosafety concerns, the utilization of polioviruses in laboratory settings is becoming increasingly restricted. This development poses a substantial challenge to the ongoing assessment of disinfectant efficacy, particularly in the context of compositions such as propan-1-ol-based or low-concentration ethanol products, which demonstrate limited effectiveness against highly resistant non-enveloped viruses such as enteroviruses. Given the persistent threat to public health posed by the re-emergence of poliovirus in under-immunized populations due to geopolitical dynamics, there is an imperative to identify a suitable surrogate virus. Such a surrogate must closely mimic poliovirus in terms of its chemical resistance and behavior under test conditions, while posing less risk in laboratory environments. The development and validation of an appropriate surrogate virus model is therefore crucial to ensure the continued reliability of virucidal efficacy testing and to maintain high standards of infection prevention.

A variety of surrogate viruses from the picornaviridae family are currently employed in different virucidal testing protocols [12], [13], [14], [15], [18], [19], [20]. Other surrogate virus candidates, particularly for water disinfection, include waterborne viruses such as picornaviruses [21]. Specifically, coxsackievirus B5 (CVB5) is classified as a genotype of the Enterovirus genus and is included in the fifth draft of the U.S. Environmental Protection Agency’s (U.S. EPA) list of drinking water contaminants (CCL5) [22]. CVB5 is also mentioned in the ECHA guidance part 2 [23]. Furthermore, the United States Environmental Protection Agency (USEPA) is evaluating adenoviruses, caliciviruses, enteroviruses and hepatitis A virus for the possibility of regulatory action [22]. The current US regulations require the removal or inactivation of 99.99% of enteric viruses by approved treatment methods. These methods are based on bench-scale studies where a specific virus is exposed to a disinfectant at various environmental conditions until reaching 99.99% (4 lg) inactivation. 

These surrogates could also have the potential to mimic the resistance characteristics of poliovirus while avoiding the associated biosafety concerns. However, Hepatitis A virus (HAV) showed a significantly higher stability in comparative studies with poliovirus, making it unsuitable as a substitute [24]. Conversely, the necessity for elevated concentrations of disinfectants would be imperative; however, the number of applicable products would be considerably limited [16]. 

The joint commission “Virus Disinfection” of the German Association for the Control of Viral Diseases (DVV) and the German Society of Viruses (GfV) has proposed yet another surrogate Minute virus of mice (MVM) from the parvoviridae family. A recent study demonstrated that the MVM is highly stable against disinfectants [25] while a further study demonstrated that GDA and PAA, the active substances employed in surface and instrument disinfectants, respectively, inactivated MVM and PV-1 in a comparable manner [16]. Parvoviruses, and consequently MVM, display an intrinsic resistance to alcohols, characteristic previously described by Eterpi in 2009 [26]. Therefore, MVM can’t be used to test the virucidal efficacy of alcohol-based hand antiseptics.

In this study, two strains of EMCV were selected for their suitability as poliovirus surrogates. A comparison was made between the resistance profiles of the aforementioned EMCV strains and that of the PV-1, the current test virus, under defined test conditions. The analysis focused on five commonly used active substances, representing different classes of virucidal agents. While commercial disinfectant products often rely on sophisticated formulations for efficacy, the present study evaluated virucidal resistance by focusing on these five agents, providing a controlled and reproducible basis for surrogate virus assessment. Ethanol, propan-1-ol and propan-2-ol are primarily used in hand antiseptics. However, previous studies on poliovirus have demonstrated that the commonly used hand antiseptics based on propan-1-ol and propan-2-ol are insufficient at the required short contact times [27], [28], [29]. Since ethanol has been shown to inactivate the poliovirus [27], [28], [30], its efficacy against EMCV and PV-1 at varying concentrations and exposure times was investigated in this study. Furthermore, two additional agents, GDA and PAA, which are employed in surface and instrument disinfection of resistant non-enveloped viruses, have been evaluated against MVM and PV-1 in a study conducted by Steinmann et al. [16]. The objective of this study was to define a surrogate virus candidate for the substitution of poliomyelitis virus for testing the virucidal activities of instrument and surface disinfection. Consequently, the efficacy of these two agents against EMCV was also evaluated in the present study.

The cultivation of both cardioviruses, EMCV-C and EMCV-U, in high titers was observed in the two test laboratories with minimal technical effort. The viruses are classified as being of safety level 2 according to TRBA 462. Hence, the safety measures that are routinely employed for their handling in the laboratory are regarded as sufficient. Generally, products containing an ethanol content of 80% (w/w) or more can, if necessary, inactivate poliovirus [27], [28], [30]. Therefore, the time kinetics tests were carried out with a non-active concentration 70% (w/w) ethanol (with 1 min contact time) [29], [30]. Given the steep kinetics of ethanol, this concentration was assumed to represent the limit of efficacy. As illustrated in Figure 1 (Fig. 1), a time dependence can be exhibited.

According to EN 14476 [17], hand antiseptics should be active in a maximum of 60 s, as longer exposure times are not applicable in practice. Therefore, the exposure time was determined to be one minute in order to investigate the influence of the ethanol concentrations on the inactivation of PV-1 and the two EMCV strains. The results for laboratory 1 and laboratory 2 confirm literature data, see Figure 2 (Fig. 2) showing that a concentration of at least 80% (w/w) inactivate poliovirus not in every case [5], [28], [30]. In laboratory 1, the same concentration is also required to inactivate the EMCV strains, whereby EMCV-U is somewhat more resistant than EMCV-C strain and demonstrates a greater degree of similarity to PV-1 (see Figure 5 (Fig. 5)). 

In laboratory 1, no significant differences were observed between the two EMCV strains and PV-1. In laboratory 2, the results obtained with EMCV-U and PV-1 were similar with those in laboratory 1. However, both viruses demonstrated slightly enhanced resistance to ethanol compared to the results observed in laboratory 1. 

Both EMCV strains were completely resistant to propan-1-ol and propan-2-ol up to a concentration of 90% (v/v) and an exposure time of 60 minutes (see Table 3 (Tab. 3) and Table 4 (Tab. 4)) and thus confirm known literature data [5], [29], [30].

In accordance to Steinmann et al. [16], two other active substances used for surface and instrument disinfection were tested for their effectiveness against PV-1 and the two EMCV strains. Therefore, both EMCV strains and PV-1 were tested with PAA and GDA analogue to the tests of the Steinmann study. By participating in the tests for this publication, the authors also have the original data with PV-1, so that they can be compared directly with the results of the cardio viruses. Very similar reductions in PV-1 and EMCV-U could be determined with GDA in laboratory 1 and 2, see Figure 4 (Fig. 4). EMCV-C shows a higher resistance to GDA than EMCV-U in both laboratories. However, a lower reduction is achieved for all three viruses in laboratory 2 compared to laboratory 1. 

The underlying causes of these variations may be attributable to the two biological systems employed: cell culture and viruses. It is plausible that the slight variations in laboratory conditions and the handling of procedures or materials, which are considered negligible, could cause an effect on these sensitive systems. Potential sources of variation may include the virus’s origin, the cell line utilized, the composition of the cell culture medium, the source of fetal calf serum, supplements, the number of passages undergone by the cells and viruses, and any other variables influencing the experimental outcome. As indicated by virus validation studies for medicinal products, small differences in the production parameters of virus stocks, such as protein content or temperature, can lead to differences in the reduction of virus infectivity by whatever mechanism [12]. Differences in the resistance of the PV-1 from the two laboratories were also found in this study. These may be due to the different origins of supply.

This effect can also be seen with PAA. In laboratory 2, a modestly higher concentration (0.08% (v/v) versus 0.075% (v/v)) is required for (complete) inactivation of the test viruses compared to laboratory 1 (Figure 3 ). The reductions due to PAA vary only marginally among the three viruses. In laboratory 1, EMCV-U shows a very similar reduction to PV-1. EMCV-C is more resistant here than EMCV-U. From these results it can be deduced that EMCV-U can also achieve reductions comparable to PV-1 with aldehydes and oxidative agents.

Limitations of EMCV as a surrogate virus candidate are as follows. In order to achieve a declaration of efficacy for surface and instrument disinfectants within the virucidal efficacy range as outlined by European standards, it is necessary to submit the results of the quantitative suspension test (phase 2/ step 1) and a practice-oriented test (phase 2/step2) to the relevant authorities or notified bodies [6]. This tiered approach is, however, not feasible for poliovirus due to high drying loss even though poliovirus is needed to demonstrate the disinfectants’ efficacy against enteroviruses and to claim virucidal efficacy [6]. Conducting a practical test with poliovirus has proven to be challenging due to its high drying loss on test carrier surfaces. According to preliminary screenings, both EMCV strains and two other picornaviruses, namely enteric cytopathic human orphan (ECHO) virus 1 strain Farouk (ATCC VR-1038) and enteric cytopathic bovine orphan (ECBO) virus, are ineffective in addressing this problem showing also a drying loss of 2–3 lg on surfaces (data not shown). This suggests that if EMCV is used as a surrogate for polioviruses, proof of virucidal efficacy against enteroviruses continues to be provided only in quantitative suspension tests. 

### Limitations

The present study is limited to data obtained from quantitative suspension tests (phase 2/step 1), which, while standardized, do not fully replicate the practical conditions of use. It has been demonstrated that picornaviruses, including the encephalomyocarditis virus (EMCV), exhibit limited resistance to drying, which precludes the use of carrier-based methods that simulate surface contamination. Moreover, due to the potential zoonotic nature of EMCV, it may be considered unsuitable for testing conducted on volunteers. This has the effect of restricting the scope for in vivo validation.

## Conclusions

The study’s findings did not indicate a clear distinction between the two cardio viruses, except for ethanol. For ethanol, the best correlation between PV-1 and EMCV-U was shown (Figure 5 ). However, it seems that the use of EMCV-U as a substitute for PV 1 may be a suitable approach in the testing of the efficacy of ethanol-based products intended for hand antisepsis and surface disinfection as well as for PAA and GDA containing products. It is important to note that the investigations presented thus far have been conducted in two test laboratories. To help define a surrogate for PV-1 in the European standards as part of the Global Poliovirus Containment Action Plan, it may be advisable to confirm the results in additional laboratories. Therefore, it would be beneficial to undertake a comparison of EMCV-U performance with PV-1 and/or other candidate test viruses in a European interlaboratory ring trial. 

## Notes

### Competing interests

The authors declare that they have no competing interests.

### Funding

This study was not funded.

### Acknowledgments

We would like to express our gratitude to Sven Reiche for the recommendation and providing of two EMCV candidate virus strains. We would like to thank Anke Herrmann, Manuela Hanisch and Carolin Benzinger for the excellent performance of the laboratory tests. We would also like to thank Dora Csertö for her support with the statistical analysis.

### Authors’ ORCIDs

- Eggers M: https://orcid.org/0000-0001-8485-9485
- Konrat K: https://orcid.org/0000-0001-9963-5222
- Hübner N: https://orcid.org/0000-0002-6095-4936
- Schwebke I: https://orcid.org/0009-0007-6708-5845

## Supplementary Material

Supplementary Table S1 Surrogate viruses for poliovirus

## Figures and Tables

**Table 1:**
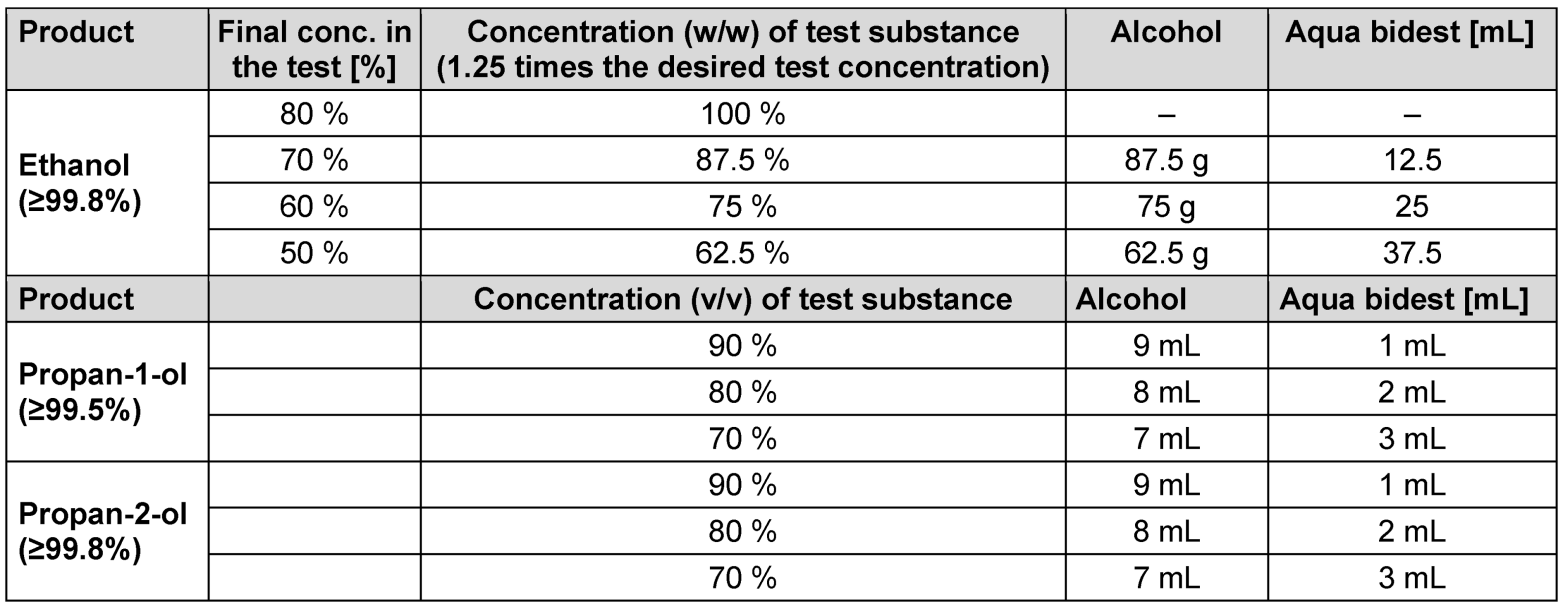
Guide for preparation of alcoholic test solutions

**Table 2:**
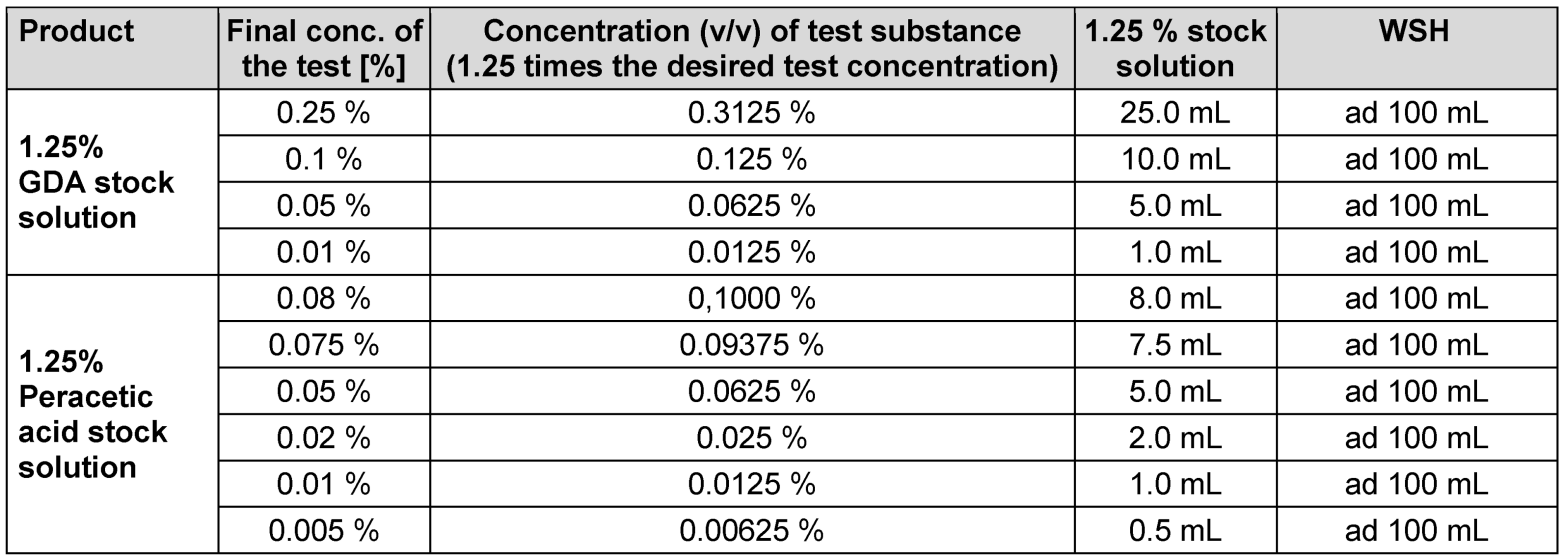
Guide for preparation of glutaraldehyde and peracetic acid test solutions (v/v)

**Table 3:**
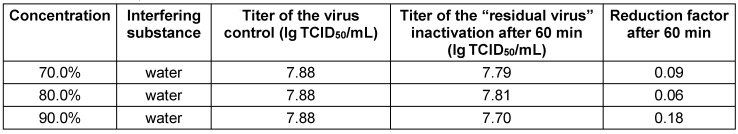
Concentration-dependent virucidal activity of propan-1-ol against EMCV-U in the quantitative suspension test

**Table 4:**
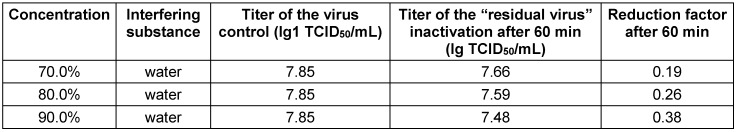
Concentration-dependent virucidal activity of propan-2-ol against EMCV-U in the quantitative suspension test
