# Supplementary material for: A potential surrogate for poliovirus in testing the efficacy of hand antiseptics according to the Global Poliovirus Containment Action Plan
Source: GMS Hyg Infect Control. 2025 Jul 9;20:Doc39. doi: 10.3205/dgkh000568 (PMC12381869; doi:10.3205/dgkh000568)
Supplement: Supplementary Table S1 Surrogate viruses for poliovirus [file HIC-20-39-s-001.pdf]

Supplementary Table S1 Surrogate viruses for poliovirus

Surrogate viruses	Isolate	***Picornaviridae***	Genus	Origin	Host cells	BSL	GMO	Virus bank	References
PVSRIPO		*Picornaviridae*		genetically modified nonpathogenic version of the oral poliovirus Sabin type 1 with the IRES from human rhinovirus type 2 (HRV2)	Vero		x		[18]
Poliovirus S19/Mah		*Picornaviridae*	*Enterovirus C*	hyper-attenuated, genetically stable carrying poliovirus capsid sequence of type 1 strain “Mahoney”	Vero	2	x	NIBSC	[19]
Coxsackievirus B5 (CVB5)	Faulkner (Kentucky/ US/52)	*Picornaviridae*	*Enterovirus B*	human	LLC-MK2 Derivative, BGM	2		ATCC VR-158	[25], [24]
Echo virus type 1	Farouk (Egypt/51)	*Picornaviridae*	*Enterovirus B*	human	LLC-MK2 Derivative, BGM	2		ATCC VR-1808	[20]
Hepatitis A virus (HAV)		*Picornaviridae*		human	FRhK-4	2		ATCC VR-1402	
Enterovirus E= enteric cytopathic bovine orphan virus (ECBO)		*Picornaviridae*	*Enterovirus E*	cattle	MDBK,<br>BT	1		ATCC VR-248	[31], [32]
Encephalomyo-carditis virus (EMCV) 1	LC 75 (Cuba)	*Picornaviridae*	*Cardiovirus*	mouse	BHK 21	2		FLI	
Encephalomyocardi tisvirus 1	Ungarn	*Picornaviridae*	*Cardiovirus*	mouse	BHK 21	2		FLI	
Encephalomyo-carditisvirus	EMC	*Picornaviridae*	*Cardiovirus*	chimpanzee	Vero	2		ATCC VR-129B	[12]
Encephalomyo-carditisvirus	Mengo vMC0	*Picornaviridae*	*Cardiovirus*	mouse<br>Genetically engineered Mengo viruses with artificial deletions in the 5′ noncoding poly(C) tracts	HeLa cells or vero cells	2	x	ATCC VR-1597/ ATCC VR-129b	[13], [14], [15], [33]
Minute virus of mice	Crawford	*Parvoviridae*	*Parvovirus*	mouse	A9	1*/2		ATCC VR-1346	[23]

*Germany

Attachment to: Eggers M, Konrat K, Hübner N, Schwebke I. A potential surrogate for poliovirus in testing the efficacy of hand antiseptics according to the Global Poliovirus Containment Action Plan . GMS Hyg Infect Control. 2025;20:Doc39. DOI: 10.3205/dgkh000568
